# Scapholunate and lunotriquetral interosseous ligament augmentation with internal bracing in perilunate dislocation: Case reports

**DOI:** 10.1097/MD.0000000000026827

**Published:** 2021-09-24

**Authors:** Soo-Hwan Kang, Seungbum Chae, Jongmin Kim, Jiwon Lee, Il-Jung Park

**Affiliations:** a Department of Orthopaedic Surgery, St. Vincent's Hospital, College of Medicine, The Catholic University of Korea, Seoul, Republic of Korea; b Department of Orthopaedic Surgery, Daegu Catholic University Medical Center, Daegu Catholic University School of Medicine, Deagu, Republic of Korea; c Department of Orthopaedic Surgery, Bucheon St. Mary's Hospital, College of Medicine, The Catholic University of Korea, Seoul, Republic of Korea.

**Keywords:** augmentation, internal bracing, perilunate dislocation, scapholunate interosseous ligament

## Abstract

**Rationale:**

The goals of surgical treatment of the perilunate dislocation (PLD) are confirmation of reduction, ligament repair, and supplemental fixation of the bony architecture. Open reduction and direct repair of the torn ligament are recommended for acute PLD. However, repair of the scapholunate interosseous ligament (SLIL) and lunotriquetral interosseous ligament (LTIL) is often unreliable, and secure repair is challenging. Internal bracing (IB) is an augmentation method that uses high-strength non-absorbable tape and enhances strength and support during the critical period of ligamentous healing. However, there is a paucity of data on the application of IB for PLD in the wrist. We report 3 cases of PLD that were augmented with IB after SLIL and LTIL repair.

**Patient concerns:**

All 3 cases were men who visited our emergency department with wrist after falling off a ladder.

**Diagnoses:**

Initial radiographs revealed a dorsal PLD.

**Interventions:**

Surgically, complete rupture of the SLIL and LTIL were confirmed. K-wires were placed into the scaphoid and lunate and used as joysticks to correct the intercalated segment instability pattern. This usually requires correcting scaphoid flexion and lunate extension and closing the scapholunate interval. Prior to SLIL and LTIL repair, temporary intercarpal fixation was performed with K-wires to maintain the carpal relationship. The dorsal SLIL and LTIL were carefully repaired using suture anchors. However, ligament repair was unreliable, and insecure. In view of the likelihood of insufficient repair, we performed IB augmentation using synthetic tape.

**Outcomes:**

At the last follow-up, all cases were pain-free and had returned to all activities. The last follow-up radiographs showed good alignment of the carpal bones and no arthritic changes.

**Lessons:**

IB augmentation can reduce the period of K-wire fixation and cast immobilization and can enable early joint motion. We believe that interosseous ligament augmentation using IB is a reasonable treatment option for PLD.

## 1 Introduction

Perilunate fracture-dislocations (PLFD) and perilunate dislocations (PLD) are uncommon but devastating carpal injuries.^[1]^ They are related to severe pan-carpal injuries that can present in the setting of high-energy trauma and occur through injuries to the surrounding stabilizing structures, such as fractures and ligament disruptions.^[2]^ It may be described using the arc methodology: greater arc injury results in fracture of the respective carpal bone or radial styloid, whereas lesser arc injury is associated with ligamentous disruption.^[3]^ The dorsal scapholunate interosseous ligament (SLIL) and volar lunotriquetral interosseous ligament (LTIL) are the strongest portions of their respective ligaments and the most important structures in PLD. We will focus on PLD, which is an acute, purely ligamentous, lesser arc injury.

The general consensus is that nonoperative treatment is rarely indicated as definitive management for PLD.^[4]^ Operative management is usually indicated because the complex intercarpal relationship is difficult to maintain with closed reduction and immobilization alone.^[5]^ Inadequate realignment of the carpus can result in carpal instability, posttraumatic osteoarthritis, advanced scapholunate collapse, and loss of range of motion.^[5]^ The goals of the surgery are confirmation of reduction, ligament repair, and supplemental fixations of the bony architecture to allow for ligamentous healing. Ligament repair, particularly SLIL repair, is important for maintaining carpal stability. The restoration of the SLIL is believed to play a significant role in achieving successful long-term outcomes.^[5,6]^ However, SLIL and LTIL repair is often unreliable, and a secure repair is challenging because they are very short and are avulsed from the bony attachment. Therefore, K-wire fixation or prolonged cast immobilization may be required to maintain reduction and prevent disruption or stretching of the repair during the healing process.^[7,8]^ Unfortunately, this can lead to stiffness and muscle atrophy of the affected arm, necessitating a secondary procedure for K-wire removal postoperatively. Moreover, if the ligament is reinjured or has failed to heal at the time of K-wire removal, re-widening of the scapholunate and lunotriquetral intervals and persistent ligamentous instability can occur.^[8]^

Internal bracing (IB) is an augmentation technique that uses high-strength, non-absorbable tape that enhances strength and support during the critical period of ligamentous healing.^[9–11]^ IB augmentation has recently been applied in orthopedics, but it has not been widely applied in the field of hand surgery. A recent biomechanical study reported that SLIL repair with IB augmentation demonstrated significantly higher strength than SLIL repair without augmentation.^[12]^ However, there is a paucity of data on the application of IB for PLD in the wrist.

We augmented 3 wrists with IB after SLIL and LTIL repair. All 3 cases were performed in a similar manner. The characteristics of the 3 cases are listed in Table 1. We describe the clinical finding, surgical technique, follow-up, and outcome in detail. Written informed consents were obtained from all patients for publication of this case report and accompanying images.

**Table 1:** Patient characteristics.

No	Sex, age	Injury mechanism	Complete torn ligaments	Combined tiny bony fracture	F/U	ROM	Return to previous job	Modified Mayo wrist score
1	Male, 62Y	Falling off a ladder	SLIL and LTIL	None	14M	F: 75° E: 70°	Yes	95
2	Male, 75Y	Falling off a ladder	SLIL and LTIL	USP	10M	F: 60° E: 65°	Yes	90
3	Male, 55Y	Falling off a ladder	SLIL and LTIL	RSP	10M	F: 65° E: 60°	Yes	90

E = extension, F = flexion, F/U = follow-up, LTIL = lunotriquetral interosseous ligament, M = months, ROM = range of motion, RSP = radial styloid process, SLIL = scapholunate interosseous ligament, USP = ulnar styloid process, Y = years.

## 2 Case presentation

### 2.1 Case 1

**Figure 1:**
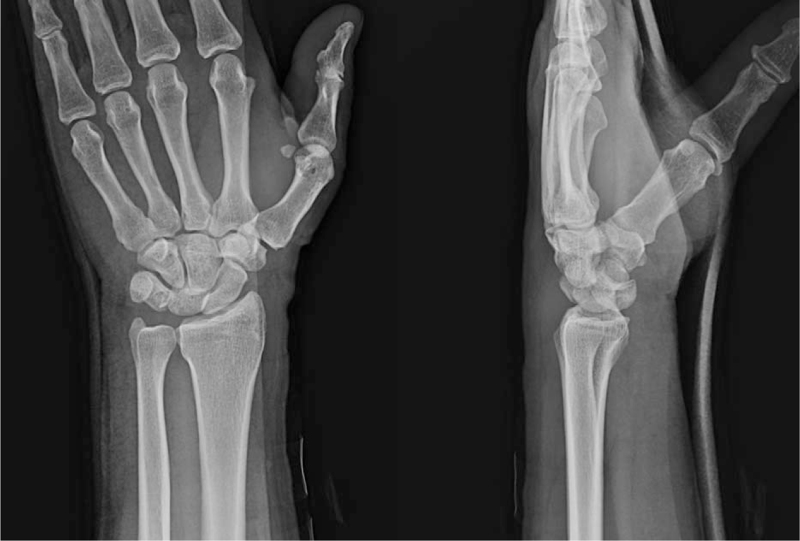
Posteroanterior and lateral wrist radiographs demonstrated a dorsal PLD. PLD = perilunate dislocation.

A 62-year-old man presented with pain in the left wrist after falling off a ladder. Initial plain radiographs revealed a dorsal PLD (Fig. 1). He had no specific medical conditions other than high blood pressure. Under general anesthesia, a 6-cm longitudinal skin incision was made on the dorsal aspect of the wrist, centered on the Lister tubercle. The incision was extended to the extensor retinaculum which was divided along the third dorsal compartment, and the extensor pollicis longus tendon was identified distally and retracted radially. The second and fourth compartments were reflected off the dorsal capsule. Elective neurectomy of the posterior interosseous nerve may be performed. The carpal bones were inspected using capsulotomy. A ligament sparing capsulotomy was done along the fibers of the dorsal radiocarpal and dorsal intercarpal ligaments.^[13]^ Bone or cartilage fragments were removed from the joint, which was irrigated to remove any hematomas or other debris.

**Figure 2:**
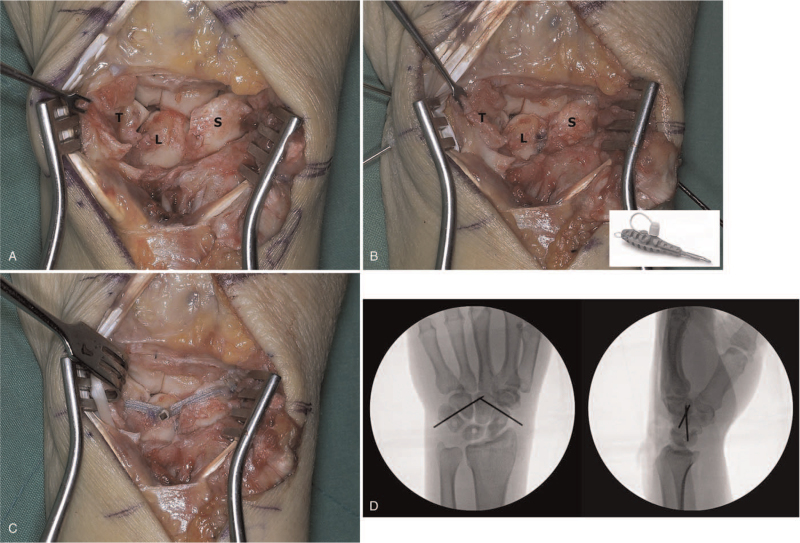
Intraoperative photographs. (A) Complete rupture of the SLIL and LTIL were confirmed. (B) The dorsal SLIL and LTIL were carefully repaired using anchor suture (right bottom: Jugger Knot Soft Anchor Suture). However, ligament repair was unreliable and insecure. (C) So we performed IB augmentation using Fibertape to secure the repair. (D) Postoperative radiographs demonstrate a well-reduced carpal alignment. L = lunate, LTIL = lunotriquetral interosseous ligament, S = scaphoid, SLIL = scapholunate interosseous ligament, T = triquetrum.

Surgically, complete rupture of the SLIL and LTIL were confirmed (Fig. 2A). K-wires were placed into the scaphoid and lunate and used as joysticks to correct the intercalated segment instability pattern. This usually requires correcting scaphoid flexion and lunate extension and closing the scapholunate interval. In most instances, the midcarpal and lunotriquetral joints are reduced, with restoration of the normal alignment of the scaphoid and lunate. Prior to SLIL and LTIL repair, temporary intercarpal fixation was performed with K-wires to maintain the carpal relationship. One was used to stabilize the scaphoid to the capitate, and the other was used to stabilize the triquetrum to the capitate. Carpal alignment and K-wire positions were confirmed using the C-arm image intensifier. The dorsal SLIL and LTIL were carefully repaired using suture anchors (JuggerKnot Soft Anchor Suture; Biomet, Warsaw, IN) (Fig. 2B). However, ligament repair was difficult, unreliable, and insecure. In view of the likelihood of insufficient repair, we decided to perform IB augmentation using synthetic tape (FiberTape; Arthrex, Naples, FL). Three 0.054″ guide wires were placed into the distal pole of the scaphoid, central portion of the lunate, and the central portion of the triquetrum. Drill holes for the IB were created using a 3.0 mm drill bit. IB augmentation was performed using suture anchors (3.5-mm DX SwiveLock SL; Arthrex), and the anchors were inserted at 90° as insertion at an obtuse angle could reduce the pullout strength (Fig. 2C). Once good alignment of the carpal bones was confirmed on the C-arm intensifier, the K-wires were buried under the skin. The capsulotomy incision was closed, and the retinaculum was repaired with interrupted absorbable 2-0 sutures. The skin was closed using nylon sutures. Postoperative radiographs demonstrated a well-reduced carpal alignment (Fig. 2D).

**Figure 3:**
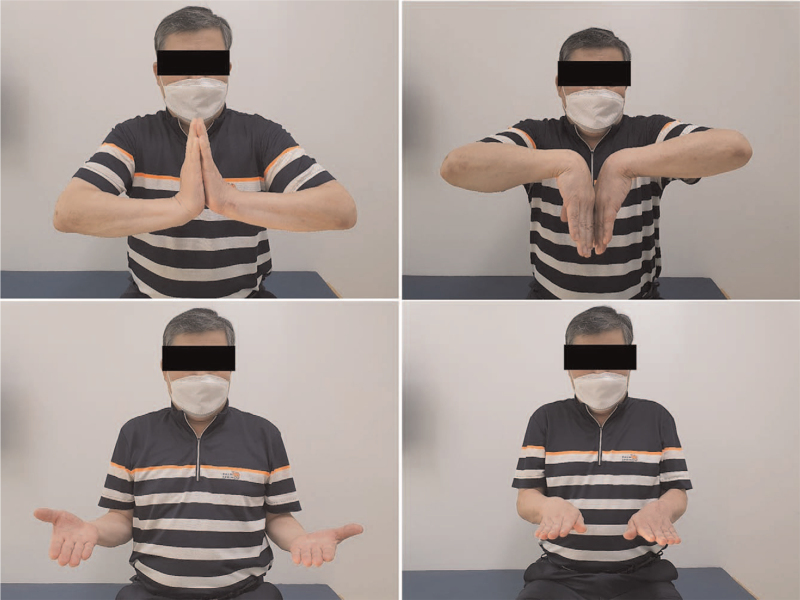
Range of motion at final follow-up.

**Figure 4:**
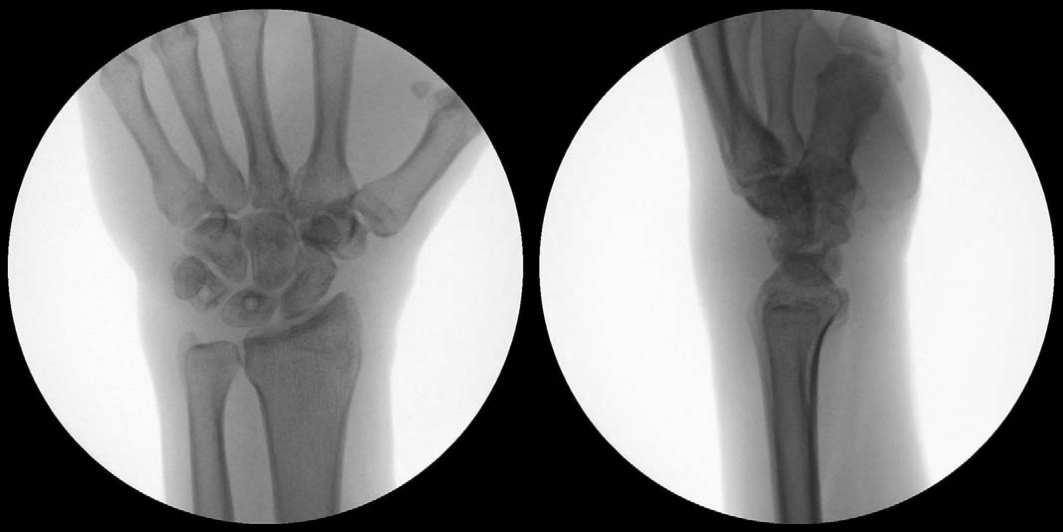
Posteroanterior and lateral radiographs at final follow-up.

A short-arm thumb spica splint was used for the first 2 weeks, and a short-arm thumb spica cast was used for the following 3 weeks. The cast and K-wires were removed 5 weeks after surgery. Rehabilitation, to restore motion and grip strength, commenced thereafter. The patient was able to return to his job 3 months after surgery. At the last follow-up, 14 months after surgery, he was pain-free and had returned to all activities. He showed excellent wrist range of motion, with flexion of 75° and extension of 65° (Fig. 3). The last follow-up radiographs showed good alignment of the carpal bones and no arthritic changes (Fig. 4).

### 2.2 Case 2

**Figure 5:**
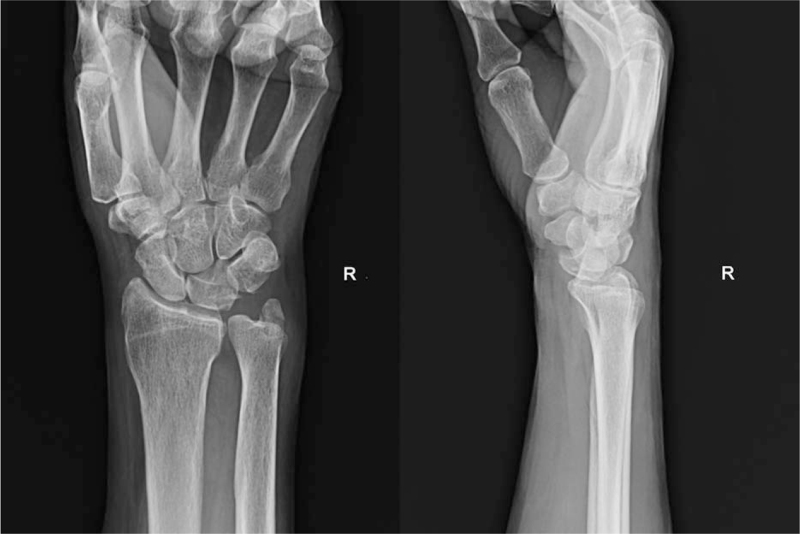
Posteroanterior and lateral wrist radiographs demonstrated a dorsal PLD and a fracture of ulnar styloid process. PLD = perilunate dislocation.

A 75-year-old man presented with his right wrist pain after falling off a ladder. Initial plain radiographs revealed a dorsal PLD and a fracture of the ulnar styloid process (Fig. 5). Concomitant injuries included fractures of the right 4th, 5th, and 6th ribs. He had no specific medical conditions other than benign prostatic hyperplasia.

**Figure 6:**
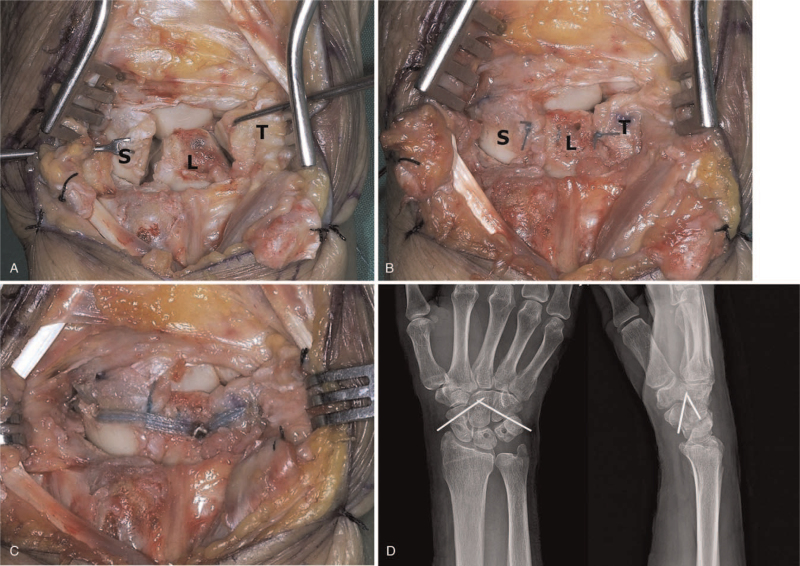
Intraoperative photographs. (A) Complete rupture of the SLIL and LTIL were confirmed. (B) The dorsal SLIL and LTIL were carefully repaired using non-absorbable sutures. However, ligament repair was unreliable and insecure. (C) So we performed IB augmentation using Fibertape to secure the repair. (D) Postoperative radiographs demonstrate a well-reduced carpal alignment. L = lunate, LTIL = lunotriquetral interosseous ligament, S = scaphoid, SLIL = scapholunate interosseous ligament, T = triquetrum.

The surgery was performed under general anesthesia. The surgical approach and method were similar to Case 1. Surgically, complete rupture of the SLIL and LTIL were confirmed (Fig. 6A). After the reduction of intercalated segment instability, temporary intercarpal K-wires fixation was performed to maintain the carpal alignment. The dorsal SLIL and LTIL were carefully repaired using non-absorbable sutures (Ethibond Excel; Ethicon INC., Bridgewater, NJ) (Fig. 6B). However, ligament repair was unreliable and insecure. Therefore, we decided to perform IB augmentation using synthetic tape as in Case 1 (Fig. 6C). Postoperative radiographs demonstrated a well-reduced carpal alignment (Fig. 6D).

**Figure 7:**
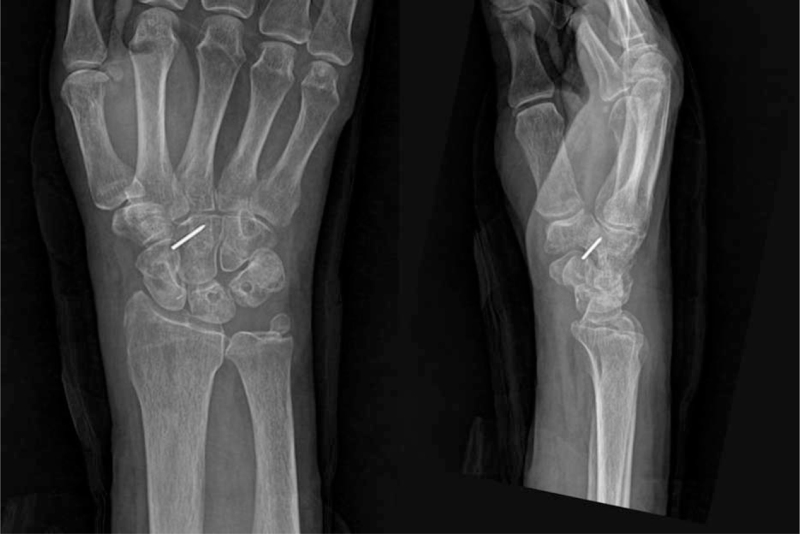
Posteroanterior and lateral radiographs at 6 months after surgery. One K-wire broke and a part of wire remained in the bone. The radiographs showed good alignment of the carpal bones and no arthritic changes.

A short-arm thumb spica splint was used for the first 2 weeks, and a short-arm thumb spica cast was used for the following 4 weeks. The cast and K-wires were removed 6 weeks after surgery. One K-wire broke and a part of wire remained in the bone, but there was no problem (Fig. 7). The patient was able to return to his job 3 months after surgery. At the last follow-up, 10 months after surgery, he showed good wrist range of motion, with flexion of 60° and extension of 65° and modified Mayo wrist score was 90. The follow-up radiographs showed good alignment of the carpal bones and no arthritic changes.

### 2.3 Case 3

**Figure 8:**
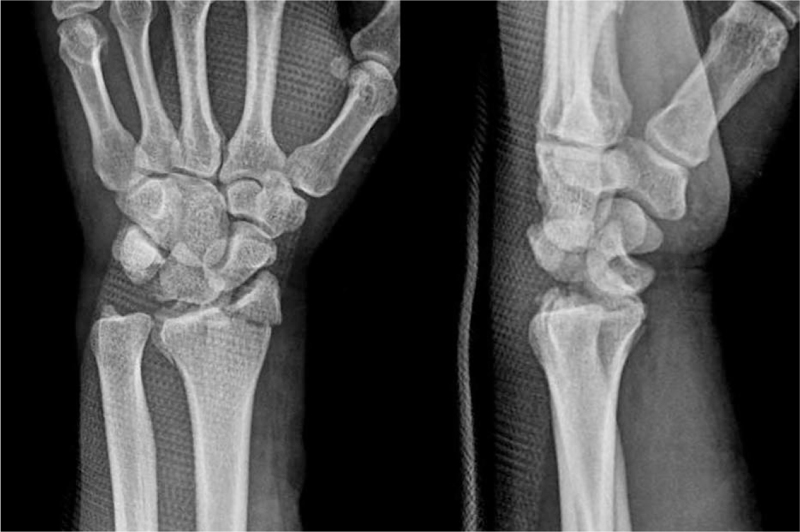
Posteroanterior and lateral wrist radiographs demonstrated a dorsal PLD and a fracture of the radial styloid process. PLD = perilunate dislocation.

A 55-year-old man presented with his left wrist pain. The patient fell off the ladder 4 days ago and was admitted via a private clinic. Initial plain radiographs revealed a dorsal PLD and a fracture of the radial styloid process (Fig. 8). He had no specific medical conditions.

**Figure 9:**
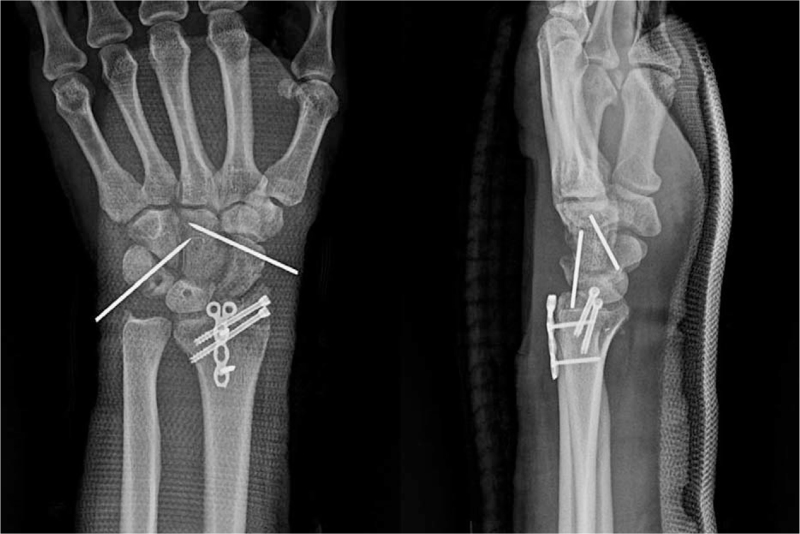
Postoperative radiographs demonstrate a well-reduced fracture fragments and carpal alignment.

The surgery was performed under axillary anesthesia. The surgical approach and method were similar to Case 1 and 2. The fracture of the radial styloid process was reduced and fixed using 2 headless compression screws (DePuy Synthes, West Chester, PA) and a mini plate. We could confirm the complete rupture of the SLIL and LTIL in the surgical field. After repair of dorsal SLIL and LTIL using non-absorbable sutures, IB augmentation with synthetic tape was performed as in Case 1 and 2. Postoperative radiographs demonstrated a well-reduced fracture fragments and carpal alignment (Fig. 9).

The postoperative immobilization method was the same as in Case 2. The cast and K-wires were removed 6 weeks after surgery. Rehabilitation, to restore motion, and grip strength, commenced thereafter. The patient was able to return to his job 3 months after surgery. At the last follow-up, 10 months after surgery, he was pain-free and had returned to almost all activities. He showed good wrist range of motion, with flexion of 65° and extension of 60° and modified Mayo wrist score was 90.

## 3 Discussion

The scaphoid, lunate, and triquetrum constitute the proximal carpal row of the wrist, which is referred to as the intercalated segment. These 3 bones are linked via intrinsic ligaments, including the SLIL and LTIL, respectively. These ligaments are anatomically divided into 3 segments: palmar, dorsal, and proximal (membranous). Biomechanical studies have shown that the dorsal SLIL is the strongest in the scapholunate joint, whereas the palmar LTIL is the strongest in the lunotriquetral joint.^[14,15]^ The dorsal component of the SLIL provides the greatest constraint to translation and rotational instability between the scaphoid and lunate bones.^[16]^ Therefore, the restoration of the SLIL is believed to play a significant role in achieving a successful long-term outcome in PLD.^[5,6]^

A variety of surgical options have been proposed for the treatment of PLD, including closed reduction and percutaneous pinning, open reduction and ligament repair, arthroscopic reduction and percutaneous pinning, external fixation, and acute proximal row carpectomy. Although closed management has been the more commonly reported treatment for PLD, the current consensus is that anatomic restoration of carpal alignment achieves better results.^[5]^ Open reduction and direct repair of the torn ligament are recommended for acute PLD in which the carpus remains reducible and arthrosis is absent.^[4,17]^ These procedures are performed because the ligament comprises tissues that are likely to heal and are strong enough to hold suture material securely during healing.^[18]^ However, reports assessing the outcomes of SLIL repair have been mixed.^[18–24]^ Inconsistencies in study design and outcome reporting have made it difficult to determine the prognosis of the procedure.^[25]^

Outcomes could be significantly improved if strength and support were enhanced during the critical time of ligament healing. IB augmentation can play this role. It offers immediate construct stability, and reduces the need for K-wire stabilization and prolonged immobilization.^[10]^ Thus, recovery can be expedited in ligament injuries, allowing earlier return to activity. Stress shielding, due to prolonged immobilization, adversely affects ligament healing, whereas excessive stress elongates the repaired ligament. However, early controlled stress optimizes the quality of collagen without the risk of lengthening after ligament repair,^[26–28]^ IB augmentation can also assist the healing of repaired ligaments in this way.

Exclusive reconstruction of the dorsal ligament may lead to excessive tightening on the dorsal side and create the risk of a hinge effect on the volar side.^[29,30]^ A biomechanical study showed that the dorsal scapholunate interval decreased after 3 different IB augmentation models compared with the intact SLIL model.^[11]^ Although the clinical significance of the volar side hinge is unclear, we consider that it will adversely affect the normal carpal alignment. Therefore, to minimize the possibility of excessive tightening on the dorsal side, we performed IB augmentation after temporary intercarpal K-wire fixation.

The attempts to permanently replace the synthetic graft for the anterior cruciate ligament (ACL) of the knee, in the past, were largely abandoned because of high failure rates.^[31,32]^ However, the IB augmentation techniques described in this paper are not homologous to artificial ligaments. In contrast to the relatively unconstrained articulation of the knee joint and intra-articular ACL, the wrist joint has highly constrained articulation and IB, which augments the repaired SLIL and LTIL, is an extra-articular structure.^[12,33,34]^ Moreover, IB augmentation is not used for permanent replacement in SLIL and LTIL but for immediate strength during ligamentous healing. It provides initial protection to a repaired ligament until maturity and revascularization occurs.^[35]^ Considering these differences, the outcome of IB augmentation in wrist joints could differ from that of the permanently replaced, synthetic ACL graft.

The present study has some limitations. Only 3 cases have been reported, and the follow-up period was not long. Therefore, we could not determine the long-term effects of IB. Nevertheless, we are unaware of any published reports on human adverse reactions to this synthetic material. Further research is needed with larger numbers of patients and longer follow-up to assess patient outcomes over time. However, we believe that this information is clinically useful for surgeons, even with these limitations.

## 4 Conclusions

IB augmentation can reduce the period of K-wire fixation and cast immobilization and can enable early joint motion. We believe that interosseous ligament augmentation using IB is a reasonable treatment option for PLD.

## Acknowledgment

The authors thank Chang Deok Weon, a medical photographer at Bucheon St. Mary's hospital, the Catholic University of Korea for help in preparing the photographs.

## Author contributions

All authors have read and agreed to the published version of the manuscript.

**Conceptualization:** Il-Jung Park, Soo-Hwan Kang.

**Data curation:** Il-Jung Park, Jongmin Kim.

**Funding acquisition:** Il-Jung Park.

**Investigation:** Il-Jung Park, Seungbum Chae.

**Methodology:** Seungbum Chae, Jiwon Lee.

**Project administration:** Il-Jung Park, Soo-Hwan Kang.

**Software:** Jongmin Kim, Jiwon Lee.

**Supervision:** Il-Jung Park, Soo-Hwan Kang.

**Validation:** Seungbum Chae, Jiwon Lee.

**Writing – original draft:** Soo-Hwan Kang, Seungbum Chae.

**Writing – review & editing:** Il-Jung Park, Soo-Hwan Kang, Seungbum Chae.
